# The Proper Planning of Store-Rooms

**Published:** 1912-09-14

**Authors:** 


### INSTITUTIONAL HOUSEKEEPING.

### The Proper Planning of Store-Rooms.

Every day hospital managers are growing less ignorant about the commissariat department, and with the advent of the trained housekeeper very general surprise is being expressed at the extent to which this important department has been, and in many institutions still is, scamped in every detail tending to promote efficiency.

In the course of an inspection extending over many years of institution premises devoted to the business of receiving, storing, and issuing supplies we have beheld dark and dreary underground cellars in which every description of food must speedily deteriorate. We have been introduced to long stone corridors lined with shelves on either hand and been bidden to admire the arrangement of dusty dry goods high over our heads. We have been ushered into big disorderly apartments cumbered with recently delivered and unsorted goods, in which all the provisions of the establishment found a refuge. In all these instances was an air of mingled neglect and hurry.

The matron ought undoubtedly to be called into counsel with the architect over the planning of the store-rooms. The amount of space which can be devoted to this most important section of the institution should be carefully mapped out into the divisions necessary to secure economy of time and space in dealing in bulk with many articles of varied kinds. The store-room in any good-sized establishment ought to contain separate compartments, each fitted with Yale lock to one key, of which duplicates must be in the hands of matron and housekeeper.

1. The Receiving Room.—This must have double doors for admitting articles in bulk and no step. It should contain an office desk, for a good deal of office work will have to be done in connection with the reception of every article dealt with in the store-rooms. There must be a good weighing-machine for heavy consignments; weights and scales for articles in lesser quantity; measuring rods for checking articles by the yard; pint, quart, bushel, gallon measures, etc.; a handy porter's truck for moving about heavy parcels; a light pair of steps; every kind of packing material; and a set of carpenter's tools. As an adjunct to the receiving room a space will be found necessary for storing packing cases, etc., either for future use, for return, or till they can be broken up for firewood.

2. The Meat Store.—This should be reserved for uncooked meat, any joints which have been left over being stored in the larder. The meat store must be on the north side and be fitted with good ventilators, ensuring a cross draught, and with wire-covered windows. It should contain a butcher's block, with chopper, saw, etc., and be hung with strong hooks instead of shelves.

3. Cheese and Bacon Store.—It is better to hang the bacon in a separate compartment to that reserved for fresh meat, as if the latter " goes off " the salted and smoked meat may suffer with it. A small meat block for cutting up sides of bacon will be convenient, and the cheese should be stored on slate slabs and be cut with rounded knives.

4. Milk, Butter, and Eggs Store.—This also must be on the north side, or shaded from sunlight by outside blinds or Venetian shutters. A double set of milk cans must be provided so that one set can undergo purification while the other is in use. Considerable care should be bestowed on the pattern of the cans so that all dirt is excluded in passage. A convenient truck must make part of the equipment so that the cans may be moved about without unnecessary clatter and fatigue. This store should be fitted with small desk and chair with lactometer, as testing the milk should make part of the ordinary dairy routine. Many processes are omitted or perfunctorily gone through for want of any convenience for recording observations. The butter should be stored on slate slabs, and should be touched as little as possible after reception. If it is required to be made into smaller pats a table covered with slate should be reserved for the process in this department, but it is better to have it sent in in pats of the required size. This store should be fitted with shelves on which pans filled with water-glass can be kept for laying down eggs in the cheap season.

5. Bread and Flour Store.—The bread must be weighed on arrival, and it will be more convenient to weigh it in its own store than in the ordinary receiving room. Hence the bakery must contain a weighing apparatus and the number of loaves must be checked every day. Strong wire shelves are best for the bread and cakes. Bins should be provided for the reception of flour.

6. General Groceries.—The sides of the room should be fitted with well-fitting bins, which will pull out, for the reception of sugar, tea, coffee, etc. Weights, scales, and measures for the issuing of stores must again form a prominent feature in this department, and it is essential that every shelf shall be readily accessible to inspection, or certain goods will moulder at the back forgotten by all.

7. The Vegetable and Fruit Store.—This should contain broad bins for the storage of potatoes, baskets and pans for other vegetables, and sets of trays to draw out, set close one above another, for storing apples, pears, etc., so that they do not touch.

8. The General Store must contain samples of every article in use in the establishment, crockery, kitchen and cleaning utensils, soap, candles, matches, and materials. Some considerable skill is needed to reduce so heterogeneous a mass of miscellaneous goods to order; but, as the list of articles required to be kept in stock seldom varies, a good system of arrangement can be practically permanent. In some establishments soap and other cleaning materials find a home of their own, and this is no doubt the more convenient arrangement. The laundry supplies will be under the direction of the laundry matron, and should be kept separate.

